# Prognostic value of systemic inflammatory factors NLR, LMR, PLR and LDH in penile cancer

**DOI:** 10.1186/s12894-020-00628-z

**Published:** 2020-05-27

**Authors:** Chen Hu, Yu Bai, Jun Li, Guoyin Zhang, Libo Yang, Chengwei Bi, Bin Zhao, Yong Yang, Ruiqian Li, Hongyi Wu, Qilin Wang, Yang Qin

**Affiliations:** Department of Urology, Yunnan Cancer Hospital, The Third Affiliated Hospital of Kunming Medical University, Kunming, 650118 Yunnan China

**Keywords:** Penile cancer, NLR, LMR, PLR, LDH

## Abstract

**Background:**

Penile squamous cell carcinoma (PSCC) represents an important public health problem for developing countries. The major prognostic factors in PSCC are pathological subtype, perineural invasion, lymphovascular invasion, depth of invasion and grade, which are hard to obtain precisely before the operation. Besides, micro-metastases will be detected in about 30% of intermediate-risk patients with clinically non-palpable inguinal lymph nodes after inguinal lymph node dissection (ILND). It means approximately 70% of patients are unable to benefit from ILND who might suffered from the complications of surgery. We hope some biomarkers could be found which are able to predict the outcome before surgery and reflect the inguinal lymph nodes metastasis.

**Methods:**

A total of 349 consecutive patients of penile cancer in Yunnan cancer hospital in China between October 2002 and December2017. Two hundred twenty-five was succeed to follow-up. The association between NLR, LMR, PLR, LDH and Overall survival (OS), progression free survival (PFS), inguinal lymph node (N stage) was analyzed with K-M analysis, univariable, multivariable logistic regression and Kendall’s tau-b correlation coefficient.

**Results:**

Multivariable analysis reveal that only PLR was significant independent factor which is associated with inferior OS and PFS; Age and LDH was associated with inferior OS; Lymph node and metastatic status remained significant for OS and PFS as NCCN and EAU Guidelines indicated; the tumor type, initial treatment and NLR LMR were not significant in predicting both OS and PFS. NLR, LMR and PLR were corresponded to N stage, while LDH was not associated with the N stage based on logistic regression model analysis. NLR, LMR and PLR were found weakly related to N stage through an application of Kendall’s tau-b correlation coefficient.

**Conclusions:**

PLR was significant independent factors for OS and PFS, Age and LDH was significant independent factors for OS. NLR, LMR, PLR was corresponded to N stage.

## Background

Penile squamous cell carcinoma has a low incidence among all cancers, according to the report there are 26,000 new cases occurring worldwide annually [1]. However, in developing countries such as Africa, Asia, South America, etc. penile cancer is still a difficult problem, where its incidence varies from 3 to 8.3 cases per 100,000 [2], especially in southwestern of China. The management of the regional lymph nodes is extremely important for long-term survival of the patient according to the National Comprehensive Cancer Network (NCCN) and the European Association of Urology (EAU) clinical practice guidelines. Both guidelines subdivide penile cancer into three groups of low risk, intermediate risk and high risk. Low-risk patients with non-palpable inguinal lymph node (cN0) can be managed by surveillance or dynamic sentinel node biopsy (DSNB), however, due to earlier Inguinal Lymph Node Dissection (ILND) surgery has a better prognosis than ILND after local lymph node recurrence, the indications for ILND recommended by the guidelines are do the operation as long as the inguinal lymph node are palpable, whether it is low-risk, moderate or high risk [3]. However intermediate-risk patients with clinically non-palpable inguinal lymph nodes, only about 30% of them are able to detect micro-metastases after ILND. That means the rest of patients approximately 70% have no benefit from ILND and suffered from the complications of surgery. So it is crucial to selected the patients who are really benefits from ILND before surgery. Because prognosis of PSCC is highly correlated with lymph nodes stage, plenty of articles have select patients through the prognostic role of biomarkers. The major prognostic factors in PSCC are perineural invasion, pathological subtype, depth of invasion, grade and lymphovascular invasion [4, 5]. These factors are hard to precisely obtain before the operation. Besides some articles used biomarkers such as p53 and squamous cell carcinoma antigen to predict inguinal lymph node positive rate and prognosis of penile cancer, but they were not applied to clinical practice [6, 7]. So we hope to find some markers that is associated with inguinal lymph nodes metastatic or can predict the outcomes.

As we known that systemic inflammatory factors play an significant role in cancer progression [8, 9]. The tumor micro-environment regulated by inflammatory cells is clearly related to cancer progression [8]. Systemic inflammatory factors are obviously reflected by changes of peripheral leukocyte, lymphocytes,neutrophils, monocytes and platelets. These factors above mentioned can reflect the tumor micro environment indirectly [8], so we collected the data of peripheral blood parameters including the neutrophil-to-lymphocyte ratio (NLR), lymphocyte-to-monocyte ratio (LMR), platelet-to-lymphocyte ratio (PLR) which have been reported to be independent prognostic factors in various of cancers [8, 10–14]. Besides, high lactate dehydrogenase (LDH) has also shown that associated with poor OS in urologic cancer [15].

Recently it has been reported that cancer patients with high NLR is associated with worse OS [16] and worse RFS and CSS [17, 18]. However, there has been no study evaluating the PLR associated with outcomes of penile cancer. In this retrospective study, we investigated the role of NLR, LMR, PLR and LDH before surgery to predicting whether micro-metastases of inguinal lymph node exist and assessed the prognostic value in penile cancer.

## Methods

We reviewed our electronic medical records system to identify men treated for penile SCC at Yunnan cancer hospital in China between October 2002 and December 2017. Experimental procedures were approved by the Human Ethics Committee of Yunnan Cancer Hospital.A total of 349 consecutive patients treated with ILND in PSCC, 225 was succeed to follow-up, Epidemiological and clinical data, including age, preoperative full blood count results NLR, LMR, PLR LDH and TNM clinical staging, tumor pathology, treatment history and oncological outcome (OS, PFS). Survival analysis was performed by the Kaplan–Meier method for univariable analysis and Cox regression method for multivariable analysis. The NLR was calculated by neutrophil-to-lymphocyte ratio, LMR was lymphocyte-to-monocyte ratio and PLR was platelet-to lymphocyte ratio which were obtained through peripheral complete blood count and blood biochemistry before the surgery. We calculate the cut-off point of the Ages, NLR, LMR, PLR and LDH according to the area under curve (AUC) given consideration to sensitivity and specificity levels.

The primary end point were overall survival (OS) and progression free survival (PFS) which defined as the time from the dates of pathological diagnosis to death and tumor progression. Univariable analysis (K-M analysis) was performed to compare the higher and lower groups of NLR, LMR, PLR and LDH. Multivariable analysis (COX logistic regression) were used to verify the individual factors respectively. *P* values less than 0.05 were considered statistical significance.

We also analyse the relation between the NLR, LMR, PLR, LDH and N stage through the logistic regression, meantime we use method of Kendall’s tau-b correlation coefficient to determine the correlation.

## Results

### NLR, LMR, PLR, LDH vs OS/PFS

A total of 225 patients had collected peripheral complete blood count and blood biochemistry before surgery. The mean (±SD) ages were 50.6 ± 13.4. The detailed data of patients are summarized in Table 1. We performed AUC curve to determine the Age, NLR, LMR, PLR and LDH cut-off value which were 53,2.94,4.74,133.5 and 188.5 respectively. The median interquartile range follow-up was 30 (16.0–63.5) months. Among the 225 patients, 171 was survival which the median interquartile range follow-up was 34 (18.0–84.0) months; 150 was disease free which the median interquartile range follow-up was 33 (18.0–85.25) months; 29 was alive with disease progression,8 was dead of other causes.

**Table 1:** Patients’ characteristics

Variable		NLR			LMR			PLR			LDH		
	All *n*=225	NLR≤2.94	NLR>2.94	p	LMR≤4.74	LMR>4.74	p	PLR≤133.5	PLR>133.5	p	LDH≤188.5	LDH>188.5	p
Age, y				0.296			0.169			0.566			0.184
<53	140	103	37		55	85		105	35		115	25	
≥53	85	54	31		45	40		55	30		65	20	
Tumor type				0.026			0.091			0.131			0.943
benign	44	35	9		17	27		38	6		35	9	
well	96	69	27		39	57		62	34		79	17	
moderate	74	49	25		35	39		52	22		59	15	
poor	8	4	4		7	1		6	2		6	2	
others	3	0	3		2	1		2	1		2	1	
Anatomic stage				0.002			0.063			0.005			0.076
0	44	33	9		17	25		36	6		33	9	
I	67	51	16		23	44		48	19		57	10	
II	39	28	11		18	21		31	8		33	6	
III	62	41	21		31	31		39	22		50	12	
IV	15	4	11		11	4		6	9		8	7	
N stage				0.001			0.024			0.009			0.053
0	148	112	36		58	90		115	33		123	25	
1	28	15	13		18	10		15	13		24	4	
2	36	26	10		15	21		24	12		27	9	
3	13	4	9		9	4		6	7		7	6	
M				0.008			0.051			0.146			0.545
0	222	157	65		97	125		159	63		179	43	
1	3	0	3		3	0		1	2		2	1	
Initial treatment				0.004			0.094			0.863			0.052
surveillance	23	10	13		14	9		16	7		15	8	
surgery	225	147	55		85	116		144	59		166	36	
Recurrence				0.01			0.635			0.004			0.235
no	150	113	37		65	85		116	34		124	26	
yes	75	44	31		35	40		44	31		57	18	
Vital status				0.000			0.001			0			0.003
Alive	171	131	40		65	106		134	37		145	26	
Dead	54	26	28		35	19		26	28		36	18	

**Fig. 1:**
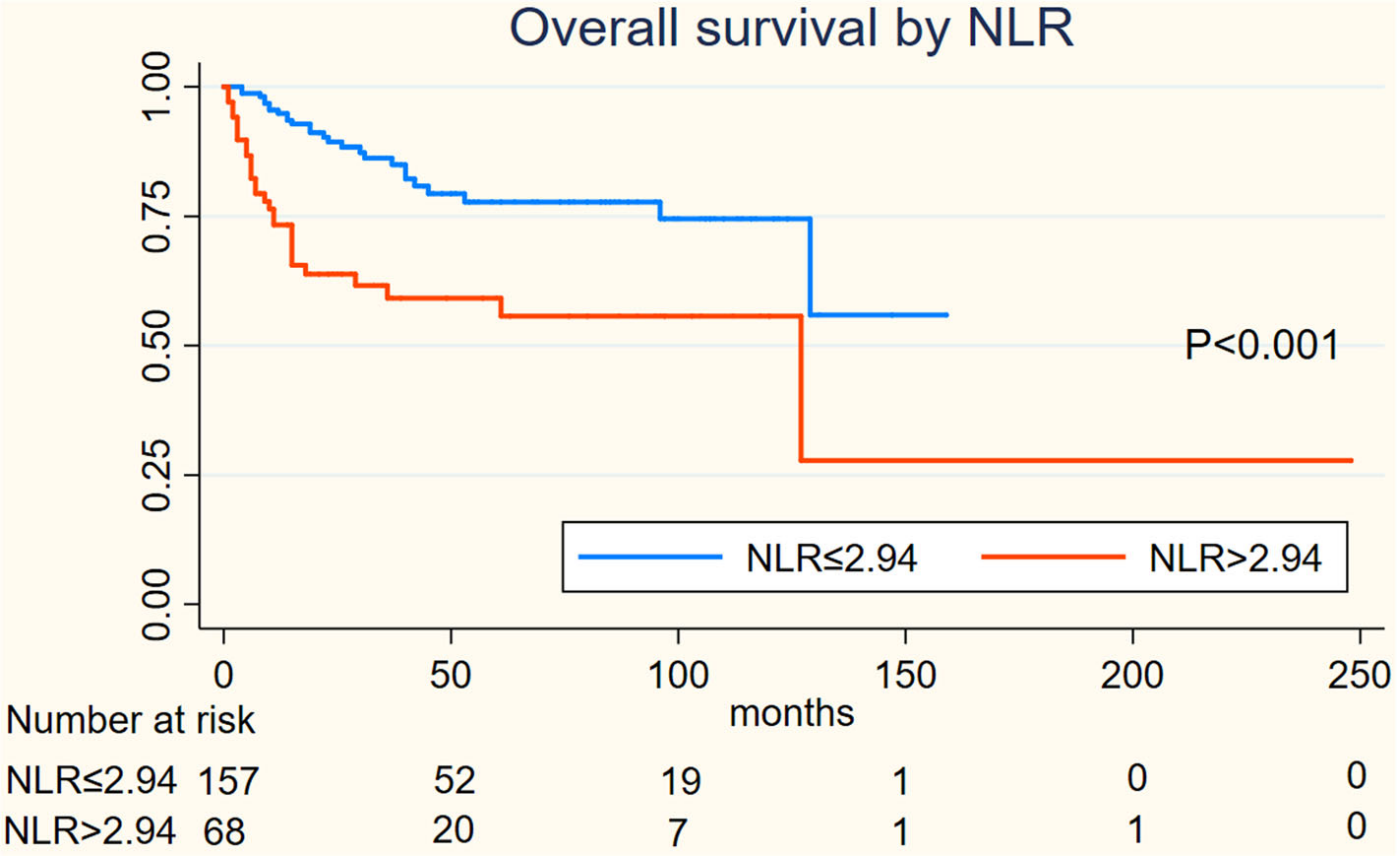
KaplanMeier curve of NLR for overall survival (OS)

**Fig. 2:**
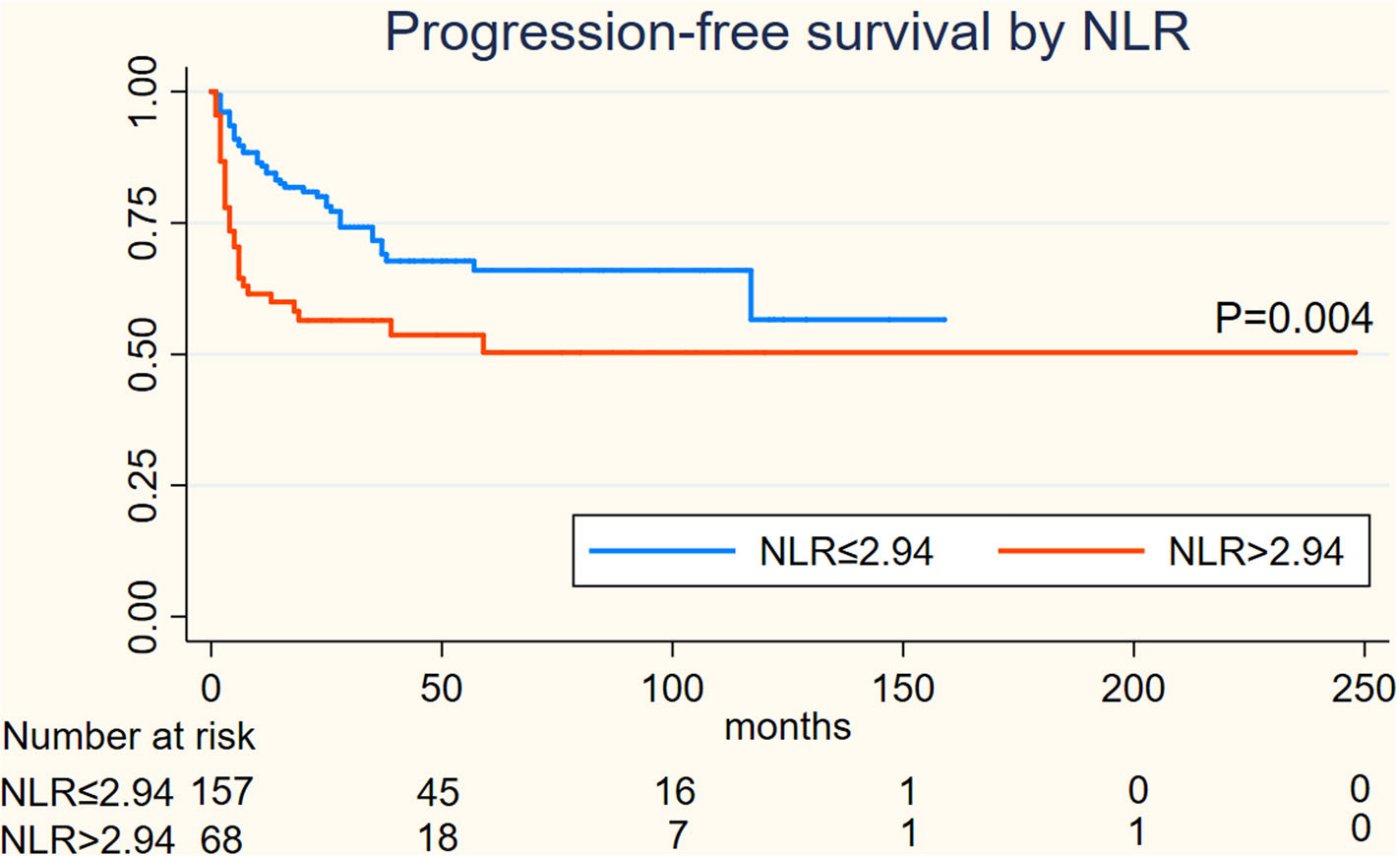
KaplanMeier curve of NLR for progression free survival (PFS)

**Fig. 3:**
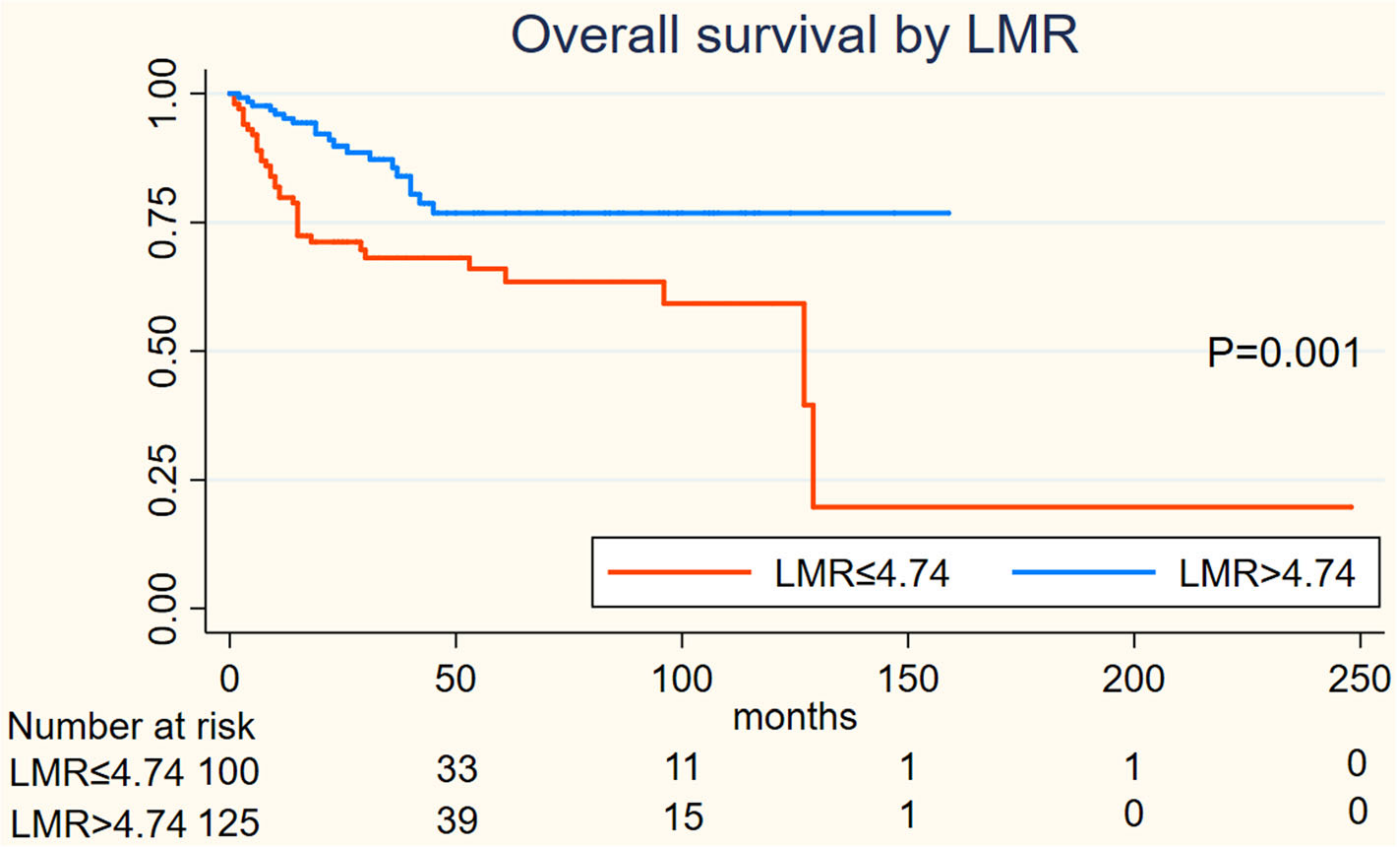
KaplanMeier curve of LMR for overall survival (OS) and 4

**Fig. 4:**
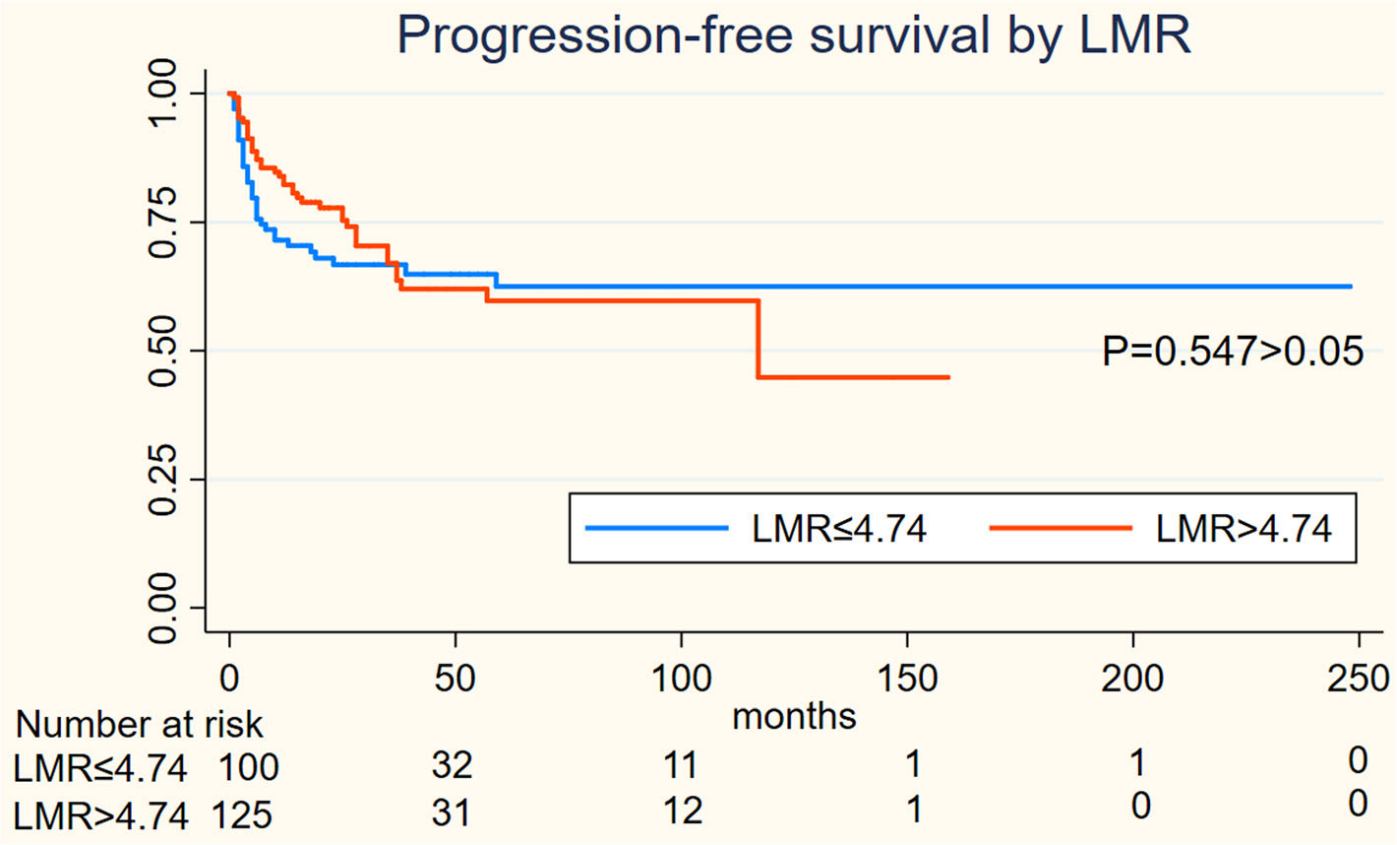
KaplanMeier curve of LMR for progression free survival (PFS)

**Fig. 5:**
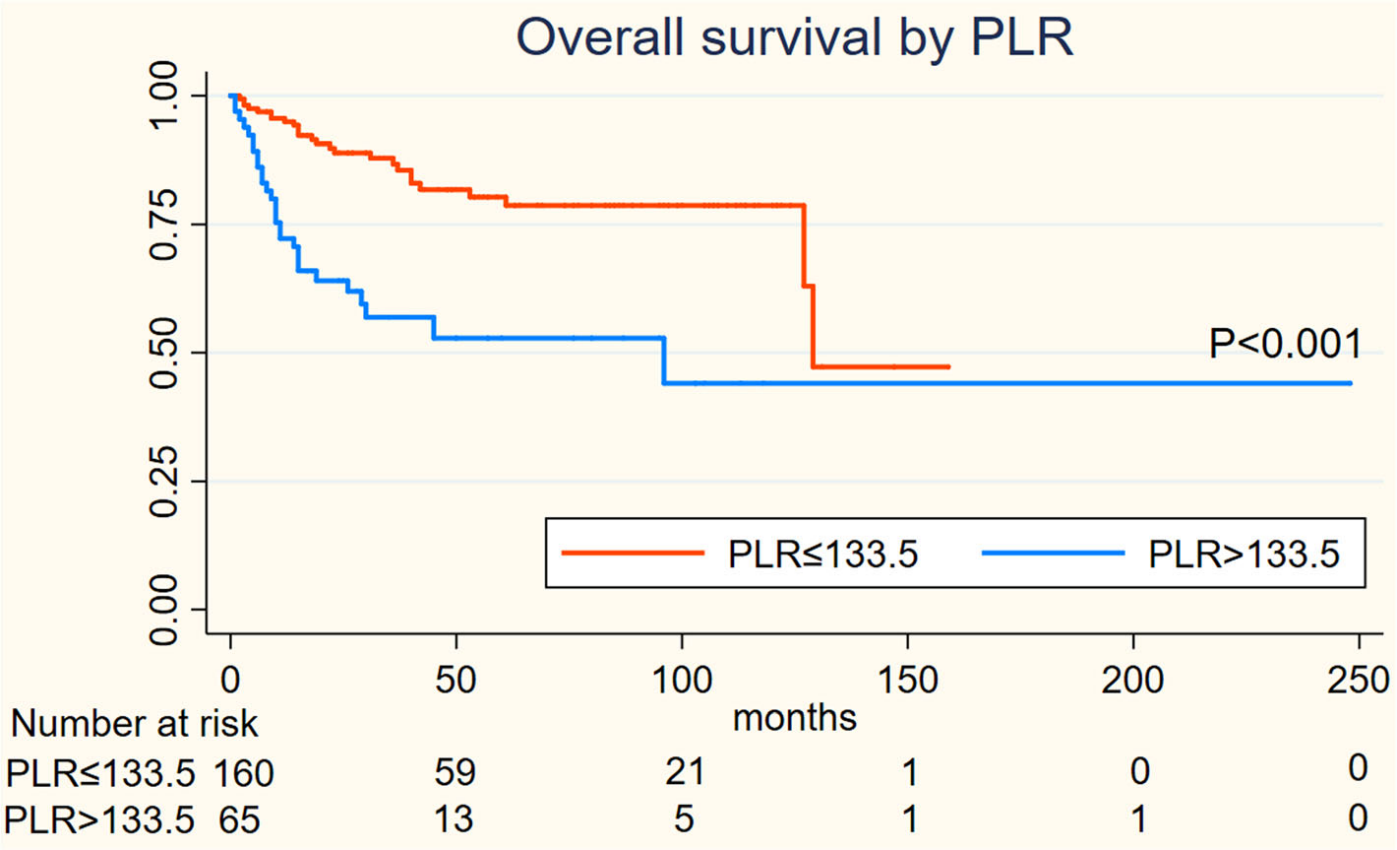
KaplanMeier curve of PLR for overall survival (OS)

**Fig. 6:**
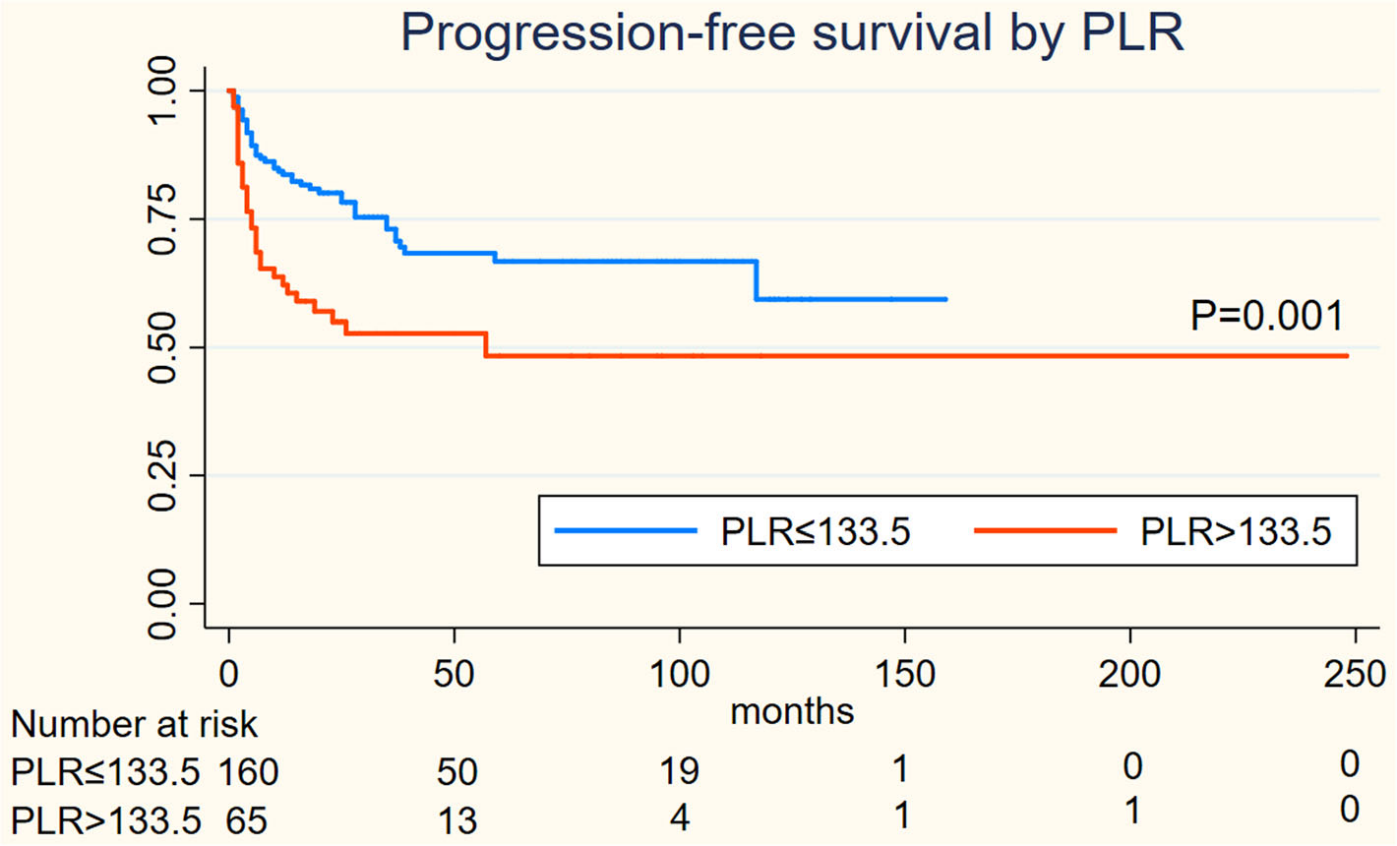
KaplanMeier curve of PLR for progression free survival (PFS)

**Fig. 7:**
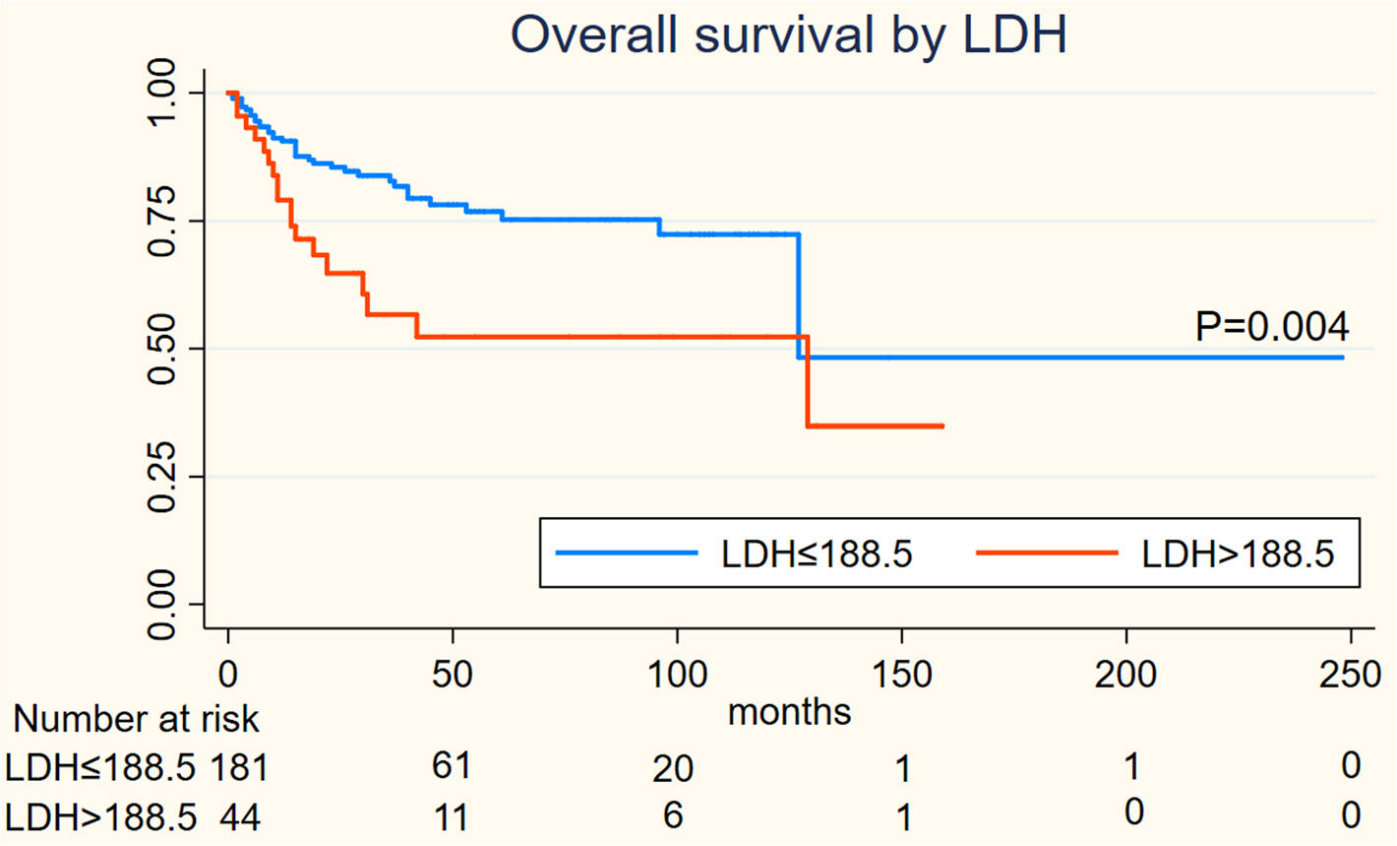
KaplanMeier curve of LDH for overall survival (OS)

**Fig. 8:**
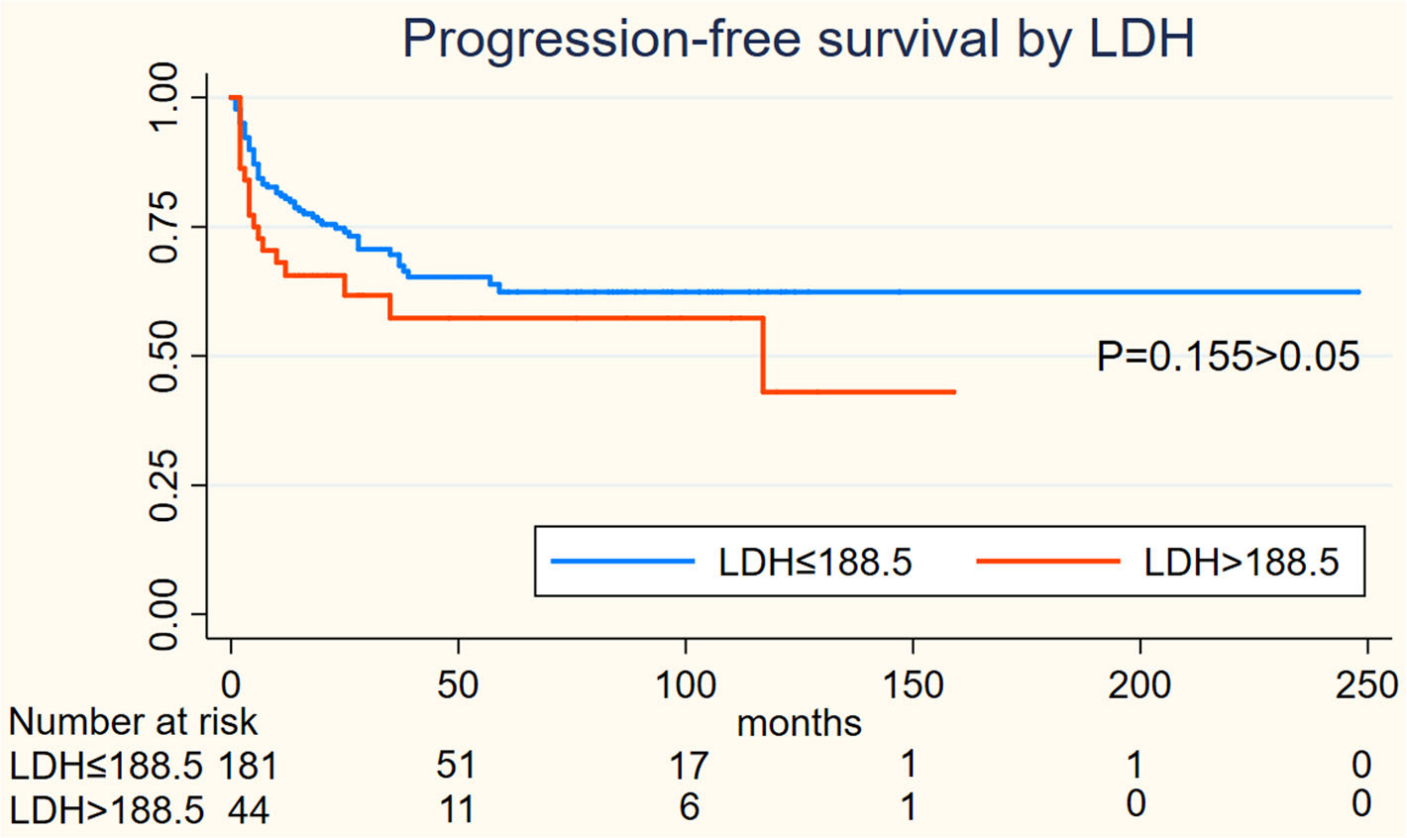
KaplanMeier curve of LDH for progression free survival (PFS)

On Kaplan-Meier analysis, NLR > 2.94 was associated with inferior OS (log-rank *p* = 0.001), and inferior PFS (log-rank *p* = 0.004) in Figs. 1 and 2; LMR > 4.74 was associated with inferior OS (log-rank *p* = 0.001), but not associated with PFS (log-rank *p* = 0.547) in Figs. 3 and 4; PLR > 133.5 was associated with inferior OS (log-rank *p* < 0.001) and inferior PFS (log-rank *p* = 0.001) in Figs. 5 and 6. LDH < 188.5 was associated with inferior OS (log-rank *p* = 0.004), but not associated with PFS (log-rank *p* = 0.155) in Figs. 7 and 8.

In univariable analyses, NLR > 2.94 was associated with inferior OS (HR = 2.967; 95% CI: 1.739–5.064, *p* < 0.001) and inferior PFS (HR = 1.944; 95% CI: 1.226–3.081,*p* = 0.005); LMR > 4.74 was associated with inferior OS (HR = 0.394; 95% CI: 0.225–0.689, *p* = 0.001) and not associated with inferior PFS (HR = 0.871; 95% CI: 0.553–1.372,*p* = 0.551); PLR > 133.5 was associated with inferior OS (HR = 3.439; 95% CI: 2.011–5.881, *p* < 0.001) and inferior PFS (HR = 2.194; 95% CI: 1.384–3.478,*p* = 0.001); LDH > 188.5 was associated with inferior OS (HR = 2.252; 95% CI: 1.267–4.002, *p* = 0.006) and not associated with inferior PFS (HR = 1.462; 95% CI: 0.859–2.487,*p* = 0.162).

We performed a multivariate analysis which reveal NLR was not significant independent factors for OS(*p* = 0.516) and PFS(*p* = 0.594); LMR was not associated with OS(*p* = 0.97) and PFS; PLR was significant independent factors for both OS(*p* = 0.001) and PFS(*p* = 0.02); LDH was significant independent factors for OS(*P* = 0.035) but not for PFS. At multivariate analysis, lymph node status (*P* = 0.03) and metastasis status remained significant for OS and PFS as NCCN or EAU Guidelines indicated; However, ages, the tumor type and initial treatment was not significant for OS and PFS in multivariable analyses **(**Table 2**).**

**Table 2:** Cox regression model for OS and PFS

Variables	OS						PFS					
	Univariable			Multivariable			Univariable			Multivariable		
	HR	95%CI	p	HR	95%CI	p	HR	95%CI	p	HR	95%CI	p
**Ages**												
<53	reference	1.077-3.174	0.026	reference	1.055-3.983	0.035	reference	0.785-1.974	0.352			
≥53	1.849			1.961			1.245					
**Tumor type**												
benign tumor	reference		0.002	reference		0.205	reference		0.006	reference		0.135
Well	0.97	0.021-0.448		0.305	0.054-1.715		0.046	0.039-0.970		0.405	0.075-2.186	
moderate	0.211	0.060-0.737		0.22	0.052-0.938		0.332	0.116-2.070		0.51	0.111-2.336	
poor	0.447	0.130-1.541		0.419	0.106-1.655		0.868	0.211-3.716		0.895	0.201-3.982	
others	0.594	0.130-2.719		0.265	0.051-1.378		0.646	0.109-3.944		0.301	0.043-2.112	
**Inguinal lymph node**												
negigitve	reference	5.243-20.918	0.000	reference	3.224-15.784	0.000	reference	3.096-8.080	0.000	reference	2.198-6.250	0.000
positive	10.472			7.133			5.002			3.706		
**Metastasis**												
0	reference	6.637-85.927	0.000	reference	2.223-40.238	0.002	reference	2.484-26.505	0.001	reference	1.184-18.072	0.028
1	23.881			9.458			8.114			4.626		
**Initial treatment**												
surveillance	reference	0.222-0.937	0.033	reference	0.18-1.006	0.052	reference	0.345-1.307	0.241			
surgery	0.456			0.425			0.671					
**NLR**												
low NLR	reference	1.739-5.064	0.000	reference	0.605-2.718	0.516	reference	1.226-3.081	0.005	reference	0.679-1.966	0.594
high NLR	2.967			1.283			1.944			1.115		
**LMR**												
low LMR	reference	0.025-0.689	0.001	reference	0.448-2.164	0.97	reference	0.553-1.372	0.871			
high LMR	0.394			0.985			0.551					
**PLR**												
low PLR	reference	2.011-5.881	0.000	reference	1.644-6.171	0.001	reference	1.384-3.478	0.001	reference	1.104-3.242	0.02
high PLR	3.439			3.186			2.194			1.892		
**LDH**												
low LDH	reference	1.267-4.002	0.006	reference	1.051-3.808	0.035	reference	0.859-2.487	0.162			
high LDH	2.252			2			1.462					

### NLR LMR PLR LDH vs inguinal lymph node-negative/positive

In logistic regression analyses, NLR (HR = 2.212; 95% CI: 1.228–3.985,*p* = 0.008), LMR (HR = 0.537; 95% CI: 0.308–0.937,*p* = 0.029), PLR (HR = 2.478; 95% CI:1.365–4.497,*p* = 0.003) was significant corresponded to N stage which divided into the Node-negative group and Node-positive groups, however the LDH (*p* = 0.165) was not associated with the N stage. Meantime we also use Kendall’s tau-b method to evaluated the correlations, NLR (Kendall’s tau-b = 0.131, *P* = 0.017) LMR (Kendall’s tau-b = − 0.109, *P* = 0.046) PLR (Kendall’s tau-b = 0.161, *P* = 0.003) were weakly correlated to the N stage. In other words, higher NLR, PLR and lower LMR was more likely to detected pathologically positive inguinal lymph node after ILND. The biomarkers of NLR LMR and PLR could predict the pathological outcomes of ILND.

## Discussion

In recent years, the role of inflammatory factors in cancer development and progression has received more attention. The tumor micro-environment may be associated with systemic inflammation. Several types of cancer have been adopt inflammatory factors in prognostic scores for predict the outcome [19] and IMDC scores of advanced renal cell carcinoma also added those factors.

Recently, an elevated ratio of peripheral neutrophils-to-lymphocytes (NLR) has been recognized as a poor prognostic indicator in penile cancer, Patients with a high NLR had significantly worse CSS [17, 18] and OS [16];Patients with a low LMR had significantly worse RFS and CSS than those with a high LMR [18].

However, there is no date about the PLR and LDH in penile cancer, this paper aims to collect data from China and conduct comparative analysis of NLR, LMR, PLR and LDH, these bio-markers are critical in guiding clinical management decisions and follow-up strategies.

The incidence of penile cancer itself is very low, so it is difficult to analyze with a large sample size. The incidence of penile cancer is relatively high in China, especially in Yunnan Province, so the sample size of this paper is relatively large.

In addition, it is difficult to judge whether there is lymph node metastasis before ILND, therefore, we also hope to assist in judgment whether there is lymph node metastasis from these indicators, so that we can choose the different surgical approach.

We acknowledge the several limitations to this study including inherent biases associated with its retrospective design, insufficient time to follow-up and population heterogeneity. Besides, more than 20% of the patients were lost to follow-up which may affect the statistical results.

We originally wanted to analyze C-reactive protein (CRP) at the same time. Since the early patients failed to perform CRP testing uniformly, the indicator failed to enter the analysis.

## Conclusion

PLR was significant independent factors for OS and PFS, Ages and LDH was significant independent factors for OS. NLR, LMR, PLR was corresponded to N stage.

## Data Availability

The datasets for the current study are available from the corresponding author upon reasonable request.

## Abbreviations

NLR: Neutrophil-to-lymphocyte ratio
LMR: Lymphocyte-to-monocyte ratio
PLR: Platelet-to-lymphocyte ratio
LDH: Lactate dehydrogenase
